# Editorial: Evolution of Signaling in Plant Symbioses

**DOI:** 10.3389/fpls.2020.00456

**Published:** 2020-04-28

**Authors:** Jeanne Marie Harris, Katharina Pawlowski, Ulrike Mathesius

**Affiliations:** ^1^Department of Plant Biology, University of Vermont, Burlington, VT, United States; ^2^Department of Ecology, Environment and Plant Sciences, Stockholm University, Stockholm, Sweden; ^3^Division of Plant Sciences, Research School of Biology, Australian National University, Canberra, ACT, Australia

**Keywords:** ericoid mycorrhiza, arbuscular mycorrhiza, symbiotic nitrogen fixation, LysM receptor, legume nodules, actinorhizal symbiosis, evolution of signaling, symbiotic signaling

Plants are surrounded by microbes, but only a small number of microbes have evolved an intimate, endosymbiotic association, in which they live inside host cells. Root symbioses are important sources of nutrition for plants and microbes alike, with over 80% of all terrestrial plants forming intracellular symbioses with arbuscular mycorrhizal fungi. This Research Topic explores the evolutionary links between different plant root endosymbioses, focusing specifically on the evolution of signaling in four: the very ancient arbuscular mycorrhizae, the more recent ericoid mycorrhizae, and root nodules formed with nitrogen-fixing soil bacteria, either with *Frankia* (in actinorhizal nodulation) or rhizobia (in rhizobium-legume nodulation) (Genre and Russo, 2016). In each case, symbiosis is initiated after signal exchange between the two partners that ultimately leads to the plant hosting the microbes inside plant cells, requiring changes in plant host development and physiology (Geurts et al., 2016; MacLean et al., 2017). During establishment of the endosymbiosis, continued signal exchange between host and microbe functions to fine-tune the interaction.

Most of the plant microbiome is found on the surface of the plant, or between plant cells. Access to the exclusive niche inside cells begins with perception of microbial signals, initiating a cascade of events within the plant host allowing infection and leading to intracellular accommodation of the endosymbiont. The arbuscular mycorrhizal symbiosis is associated with the colonization of land by plants, thus recognition of mycorrhizal fungi is ancient and widespread throughout the plant kingdom. Curiously, the plant signaling pathway triggered by mycorrhizal fungi is also used by bacterial newcomers to the plant endosymbiotic world, rhizobia and *Frankia* strains. Rhizobial signal molecules are almost identical to mycorrhizal signal molecules: short chains of chitin with a lipid tail, lipochitooligosaccharides (LCOs). The *Frankia* and ericoid mycorrhizal signal molecules are unknown, but the signal of most *Frankia* strains is almost certainly not an LCO Cissoko et al..

Recent key studies have overturned our understanding of the evolution of root endosymbiosis and put this special issue in context: in particular the conclusion that the most likely scenario is a single gain of root nodule symbiosis in a clade of Rosids, followed by multiple losses of the symbiosis (Griesmann et al., 2018; van Velzen et al., 2018). This likely involved the symbiont *Frankia* as the initial symbiont of actinorhizal species, with the later emergence of rhizobia in legume symbioses (van Velzen et al., 2019). Secondly, the common use of orthologous nodulation genes across actinorhizal and legume species underlines the similarity between signaling pathways in legume and actinorhizal symbioses, which has also, at least partly, evolved from mycorrhizal symbiosis signaling (Markmann and Parniske, 2009). Contributions to this special issue have provided new insights into aspects of the evolution of root endosymbiosis by examining the similarities at all stages of symbiosis, starting with signal perception, signal transduction, control of defense responses and eventually maintenance of the symbiosis.

Both LCOs and chito-oligosaccharides (COs) are perceived by LysM receptor kinases. The history of this gene family reveals repeated gene duplications, neofunctionalization and losses. Over this is laid the shift in function from perception of short COs to mycorrhizal signals and then to rhizobial Nod factors, analyzed in this issue by Gough et al. Further, they explore the relationship between symbiotic and immune signaling. This topic is examined in more depth by Liao et al., who demonstrate subfunctionalization after gene duplication within the LysM receptor family, specifically focusing on the evolution of the tomato chitin receptor CERK1, where chitin and mycorrhizal signal recognition is partitioned among four paralogs. LysM receptors require a co-receptor to transmit signals to the nucleus, and in plant endosymbioses this function is taken by SYMRK (SYMBIOSIS RECEPTOR KINASE). Li et al. examine this next step in the pathway to ask how SYMRK transitioned from a key position in the mycorrhizal signaling pathway to a shared function in mycorrhization and nodulation. These studies clearly highlight the evolution of signal perception from an ancient chitin and mycorrhiza perception system to the more recent perception of rhizobia by legumes.

Understanding of the *Frankia*/actinorhizal root nodule symbiosis has lagged behind the study of other root endosymbioses: the signal molecule is not an LCO, many *Frankia* species are unculturable, most plant hosts are woody perennials, and culturable *Frankia* strains (a filamentous bacterium) are difficult to grow and transform. However, actinorhizal root nodules and rhizobium-induced root nodules share an evolutionary origin in that all hosts belong to a single clade within the Rosids. Taking advantage of these parallels, comparative transcriptomics and developmental studies have provided great insights into actinorhizal nodule development and function. In legumes, flavonoids signal the rhizobial partner to induce the expression of nodulation genes and synthesis of the Nod factor signal molecule, an LCO. To explore possible new structures and roles of flavonoids in actinorhizal symbioses, Gifford et al. demonstrated synthesis of flavonoids, suggesting a functional role during actinorhizal symbiosis. This will form the basis of identifying flavonoids that may play similar signaling functions between actinorhizal hosts and *Frankia* as has been described for legume-rhizobia interactions. The diversity of flavonoids provides an additional layer of specificity to the rhizobium-legume interaction that is determined by Nod factor structure, ensuring that only specific host/microbe pairs proceed to infection.

An evolutionary assessment of the various mechanisms conferring specificity is presented by Wang et al. who also highlight recent advances in our understanding of how immune responses have been co-opted to delimit successful symbiotic responses. Even successful infections must be limited, so that they do not excessively drain plant resources. Plant peptides of the CLE (CLAVATA3/EMBRYO SURROUNDING REGION-RELATED) family have long been known to control nodule numbers as part of an internal regulatory mechanism termed autoregulation of nodulation. In their review of mechanisms of autoregulation in plant-microbe interactions more broadly, Wang et al. present the first evidence that one of the components of the autoregulation of nodulation pathway, the peptide receptor CLV2 (CLAVATA2), which also controls shoot apical meristem formation, is additionally required for autoregulation of mycorrhization in the non-legume tomato. This finding further underscores the parallels between signaling between plant host and endosymbiont in the mycorrhizal symbiosis and the rhizobium-legume symbiosis.

Once inside plant cells, endosymbionts and their plant host continue to signal each other to coordinate metabolism and development. Kereszt et al. present a broad overview of the diverse functions and evolution of plant signaling peptides in symbiotic processes in legumes and non-legumes with an emphasis on families of nodule-specific cysteine-rich (NCR) peptides (over 700 in *Medicago truncatula*!) with the ability to control bacteroid differentiation and elongation. Salgado et al. find that small defensin peptides produced by *Alnus glutinosa*, also produced by other actinorhizal plants, play parallel roles to NCR peptides in nodule symbiosis, suggesting convergent evolution or a common origin of microsymbiont control mechanisms.

In addition to the mycorrhizal origins of the plant-microbe interactions shared by all these root endosymbioses, root nodule formation involves the formation of a new organ. Mycorrhizae in general do not form a new organ, so nodule organs must have a different origin, meaning that the evolution of root nodule symbioses required both the co-option of the mycorrhizal signal response pathway as well as a second evolutionary event leading to the formation of a new organ. For decades, the model for the second event was that the ancestor of the fabids (Fabales, Fagales, Rosales and Cucurbitales) acquired a predisposition for nodulation, followed by multiple gains of the ability to nodulate (Doyle, 2016). However, as mentioned above, recent phylogenomic studies demonstrate a single origin of nodulation, followed by multiple losses, thus overturning this long-standing paradigm (Griesmann et al., 2018; van Velzen et al., 2018). This raised the importance of comparative studies including non-legume root nodule symbioses in order to distinguish between common vs. lineage-specific traits essential for nodule development and function. In this issue, five studies deal with root nodule symbioses of the Rosales. Van Zeijl et al. describe the analysis of CRISPR-Cas9 mediated knockouts of four putative nodulation-related genes in *Parasponia andersonii* (Cannabaceae, Rosales), the only non-legume able to enter a root nodule symbiosis with rhizobia, uncovering a novel role for the ethylene signaling component EIN2 in intracellular infection of *P. andersonii* nodules. Billault-Penneteau et al. describe the first step to examine another root nodule-forming member of the Rosales, establishing the sister species *Dryas octopetala* (ectomycorrhizal, non-nodulating) and *Dryas drummondii* (arbuscular mycorrhizal, nodulating) as model systems. Salgado et al. compare the nodule transcriptomes of two actinorhizal species that interact with closely related microsymbionts, *Ceanothus thyrsiflorus* (Rhamnaceae, Rosales) and *Datisca glomerata* (Datiscaceae, Cucurbitales). As mentioned above, Gifford et al. analyze the role of flavonoids in the symbiosis of *D. glomerata*. Last but not least, Cissoko et al., using the best-examined actinorhizal model system, *Casuarina glauca* (Casuarinaceae, Fagales) show the similarity between Root Hair Deforming Factor and the *NIN* activating factor, with *NIN* encoding a central transcription factor essential for root nodule symbioses. This provides an important step toward the identification of the signal factor of Fagales-infective *Frankia*.

Continuing this approach, Battenberg et al. perform a comparative transcriptomic analysis of the same two actinorhizal nodulators analyzed by Salgado et al., *Ceanothus thyrsiflorus* (Rhamnaceae, Rosales) and *Datisca glomerata* (Datiscaceae, Cucurbitales), providing new support for the two-step process of nodule evolution, in which an initial event at the base of the Nitrogen-Fixing Clade is followed by additional selection pressure at the base of each host lineage. This extends the model of a single origin of nodulation (Griesmann et al., 2018; van Velzen et al., 2018), revealing additional insight into the origins of nodulation (Parniske, 2018).

Finally, the more recent ericoid and ectomycorrhizal symbioses, which evolved from fungal lineages distinct from those that form arbuscular mycorrhizae (Wang and Qiu, 2006), are revealing novel ways in which fungi can signal the plant. The ericoid mycorrhizal symbiosis in particular, is unusual in that it also is able to enter cells to form an endosymbiosis but the signaling between fungus and plant is completely unknown. Casarrubia et al. identify a small, secreted protein, OmSSP1, from an ericoid mycorrhizal fungus, that shares features with hydrophobin effectors produced by ectomycorrhizal fungi, but appears to function in ericoid infection or colonization of the *Vaccinium myrtillus* root, thus identifying one of the first molecular signals between endosymbiont and host in this symbiosis.

By comparing the conserved and divergent processes by which these microbial and plant partners interact to form endosymbioses, these research articles, perspectives and reviews, provide insight into the evolutionary history of symbiotic signaling and pave the way for a deeper understanding of the commonalities and the unique features among these endosymbiotic interactions of plant roots.

## Author Contributions

All authors listed have made a substantial, direct and intellectual contribution to the work, and approved it for publication.

## Conflict of Interest

The authors declare that the research was conducted in the absence of any commercial or financial relationships that could be construed as a potential conflict of interest.
